# Effects of the Size, the Number, and the Spatial Arrangement of Reactive Patches on a Sphere on Diffusion-Limited Reaction Kinetics: A Comprehensive Study

**DOI:** 10.3390/ijms21030997

**Published:** 2020-02-03

**Authors:** Changsun Eun

**Affiliations:** Department of Chemistry, Hankuk University of Foreign Studies, Yongin 17035, Korea; ceun@hufs.ac.kr

**Keywords:** diffusion-limited reaction, rate constant, diffusion-controlled reaction, curvature, kinetics, finite element method, Berg-Purcell model, competition

## Abstract

We investigate how the size, the number, and the spatial arrangement of identical nonoverlapping reactive patches on a sphere influence the overall reaction kinetics of bimolecular diffusion-limited (or diffusion-controlled) reactions that occur between the patches and the reactants diffusing around the sphere. First, in the arrangement of two patches, it is known that the overall rate constant increases as the two patches become more separated from each other but decreases when they become closer to each other. In this work, we further study the dependence of the patch arrangement on the kinetics with three and four patches using the finite element method (FEM). In addition to the patch arrangement, the kinetics is also dependent on the number and size of the patches. Therefore, we study such dependences by calculating the overall rate constants using the FEM for various cases, especially for large-sized patches, and this study is complementary to the kinetic studies that were performed by Brownian dynamics (BD) simulation methods for small-sized patches. The numerical FEM and BD simulation results are compared with the results from various kinetic theories to evaluate the accuracies of the theories. Remarkably, this comparison indicates that our theory, which was recently developed based on the curvature-dependent kinetic theory, shows good agreement with the FEM and BD numerical results. From this validation, we use our theory to further study the variation of the overall rate constant when the patches are arbitrarily arranged on a sphere. Our theory also confirms that to maximize the overall rate constant, we need to break large-sized patches into smaller-sized patches and arrange them to be maximally separated to reduce their competition.

## 1. Introduction

In reaction kinetics, competition effects have been a central theme in understanding the details of reaction processes and in calculating accurate physical quantities, such as rate coefficients [1]. For example, in a bimolecular reaction between reactants A and B, if there is more than one molecule of A, there is competition among the molecules of A for a molecule of B. Specifically, in this example, because any molecule of A has the possibility to react with a molecule of B, in principle, all those possibilities should be addressed in considering the kinetics. This consideration should also be noted for a reactant that has multiple reaction sites, which can be observed in many chemical systems. For example, some enzymes have multiple active sites, and the active sites compete with each other for a substrate [2,3]. Another example is a cell surface with multiple receptors for ligands [4]. Moreover, recently, an enzyme with multiple active sites was created by rational engineering [5], which implies that the number of examples will likely increase in the future. 

The competition among reactive sites on a reactant molecule (or among reactant molecules) substantially affects the overall reaction kinetics. However, it is not easy to determine the strength of the competition effect, because the strength is influenced by the complicated interplay among many factors, such as the spatial distribution of competing reaction sites over a molecule; the density, size, and reactivity of reaction sites; and the accessibility to reaction sites. Therefore, depending on the molecular properties of the reaction sites, the kinetics could be significantly different from reactant molecule to reactant molecule. Thus, many theoretical studies have focused on general principles using simple models rather than on cases of particular reactant molecules because of difficulty in obtaining the aforementioned molecular properties. The simple models used in theoretical studies are designed to be simple yet capture the essential physics; for example, a reactant molecule can be approximately modeled as a sphere that has the same volume as that of the molecule. The advantages of using such models are that we can easily understand the fundamental aspects of physics without the complications associated with real systems, and we can extend simple models to understand a particular real system by incorporating the relevant molecular features into the models. Therefore, a mathematically well-defined and physically interesting model can be effectively applied to specific chemical systems and provide a general understanding of the corresponding processes. For example, the simple reaction models proposed by Berg and Purcell have been used to understand the physics of chemoreception in microorganisms [4]. In this work, we also focus on a discussion of simple reaction models, which are explained in detail later, and these models may be applicable to many interesting phenomena in real systems, such as the effect of the distribution of receptors on a cell in signal transduction. 

In fact, to better understand the competition that occurs in reactions, many theoretical studies have been conducted [6,7,8,9,10,11,12,13,14,15,16,17,18,19,20,21,22,23,24,25]. The models used in these studies include multiple spherical reactants (sinks) [8,9,10,14,15,16,20,23,24,25] and two nonoverlapping reactive patches on a sphere [11,12,13,21]. A common feature of these studies is that, as competing reactants or competing reactive patches are moved away from each other, the overall rate constant increases. Therefore, in an infinite space, to maximize the overall rate constant, we can simply put the competing reactants in infinite separation. However, in a finite space where the separation distance between reactants is limited or upper bounded, we cannot arrange competing reactants in infinite separation, and thus, we cannot completely remove the competition between reactants. Therefore, in a confined space or on a restricted surface area, we can speculate that the key to achieving the maximal overall rate constant is to arrange the reactants or the reactive patches in a way that they are maximally separated under a given constraint. In particular, the latter case of a restricted surface area arises in a model of N reactive circular or curved disk-like patches on a sphere, which can be considered a simple model for ligand binding on a cell surface [4]. This model, called the Berg-Purcell (BP) model, and its variant models have been studied with a various number of N patches [4,26,27,28,29,30,31,32,33,34,35,36]. In fact, the competition-minimization problem in this N-patch model is similar to the one in finding the spatial arrangement of N electrons (or electron pairs) on a sphere in the Thomson problem [37,38] (or valence shell electron pair repulsion (VSEPR) theory [39]) giving the minimum potential energy. A more relevant minimization problem would be the geometric optimization problem called the Tammes problem, which is a packing problem of circles on a sphere for finding the arrangement of circles that maximizes the minimal distance between circles [40,41]. 

For an arbitrary number N of identical circular reactive patches on a sphere, it is intuitively appealing that the patch arrangement giving the maximal reaction rate is the one with maximally separated patches from each other, as in the Tammes problem. However, it is mathematically challenging to prove this proposition rigorously for any N. Even in the Tammes problem, the general solution for arbitrary *N* is not known, and solutions for a limited number of N are available. From the studies of the Tammes problem, the known solutions for the arrangements of patches for N = 2, 3, 4, 6, and 12 have very symmetrical structures, and they are the opposite ends of a diameter, an equilateral triangle in the equator plane, a regular tetrahedron, a regular octahedron, and an icosahedron, respectively [41]. In fact, in previous studies of two patches on a sphere [13], it was shown that the solution for N = 2 is that the two patches are arranged at the two opposite poles of the sphere, which is equal to the Tammes solution for N = 2. To see if other solutions of the Tammes problem are also the solutions for the cases of reaction, we investigate the cases of N = 3 and 4 using numerical methods by calculating the rate constants for various arrangements deviating from an equilateral triangle in the equator plane and regular tetrahedron, respectively. Additionally, this investigation allows us to study the nature of the variations in the rate constant due to the patch arrangement, as well as the maximum value of the overall rate constant.

For the symmetric arrangements that we expect to give the maximum values of the overall rate constant, Northrup performed a series of Brownian dynamics (BD) simulations for given numbers of identical patches and compared the rate constants from the symmetric arrangements with those from random arrangements [26]. From this comparison, it was found that the rate constant from a symmetric arrangement corresponds to the maximum value of the rate constant obtained from various random arrangements, within the statistical errors. Another interesting finding is that the ensemble-averaged rate constant from random arrangements is very close to the rate constant from a symmetric arrangement, which means that the values of the rate constant are narrowly distributed near the maximum value. Note that in this Northrup system, the patch size was fixed, and the size of a disk-like patch (or spherical cap) was relatively small in the sense that the rate constant of a single patch on a sphere could be approximated by the rate constant of a disk in a plane (or the Hill rate constant [42]). Therefore, the main variable determining the rate constant in the Northrup system was not the spatial arrangement nor the size of the patches, but the number of patches.

Instead of fixing the patch size, Lu fixed the fraction of total reactive patch area over the entire surface area, while the patch size was free to change [29]. In this study with BD simulations, random arrangements were used, and the rate constant was calculated from the ensemble-averaged value of the arrangements. One interesting finding from the study is that, as the patch size decreases, the rate constant increases and eventually can reach the Smoluchowski rate constant, which is obtained when the whole surface of the reactant is fully reactive [1,43]. Additionally, Wu and Lu investigated how the spatial distribution of patches, characterized by a quantity called the separation index, affects the overall reaction kinetics [30]. They defined the separation index as a measure of relative patch-patch distance. In that study, they used two sizes of patches, which was the same size used in the original Northrup system and a size whose patch area was 25 times larger than the area of the Northrup patch. For each case of patch size, they generated identical patches randomly distributed on a sphere, calculated the overall rate constant for each patch distribution, and obtained the distribution of the rate constants. Interestingly, they found that the distribution of the rate constants was very narrow for both cases, which is consistent with Northrup’s narrow distribution. However, they did not directly compare the ensemble-averaged rate constant with the rate constant from symmetric arrangements, as in Northrup’s work. For this comparison, we employ the kinetic theories that provide us with the formulas for the rate constant for symmetric or uniformly distributed patches. 

In fact, in addition to numerical studies based on BD simulations, theories explaining the kinetics for N patches on a sphere have been developed for evenly or uniformly distributed patches, and these theories can also be practically applicable to cases of randomly distributed patches. A theoretical study on this subject was initially carried out by Berg and Purcell [4] with their famous Berg-Purcell model. They assumed that the patches were evenly distributed over a sphere and derived a simple formula for the overall rate constant as a function of the patch size, the number of patches, and the radius of the sphere. Later, Shoup and Szabo derived the same formula from an idea that a sphere partially covered with reactive patches can be effectively replaced by a uniformly partially reactive sphere of the same size [31]. After that, Zwanzig derived an improved formula based on an effective medium approximation, [32] and the formula gave better agreement with the BD simulation results of random patch arrangements by Northrup [26]. With the help of BD simulations of random patch arrangements, Berezhkovskii et al. proposed another formula based on the boundary homogenization (BH) theory [33,34]. Interestingly, using a mathematical approach named the matched asymptotic analysis, Lindsay, Bernoff, and Ward (LBW) obtained a general formalism of the rate constant for an arbitrary patch arrangement, from which a simple formula can be derived for a uniform patch distribution [35]. In addition to these formulas, we recently obtained another new formula [27] by incorporating the curvature effect of patches [44] and the asymptotic behavior of the rate constant near σ~1 [34] into the Zwanzig formalism [32]. We showed that, overall, our formula and the BH formula give better results than others in a comparison of all the formulas except the LBW formula in the Šolc-Stockmayer, curved reactive surface (CRS), and BP models, although the Zwanzig formula only gives the best agreement with the BD simulation performed by Northrup for the BP model [27].

One reason for the improvement in our and the BH theories is taking into account the curvature effect explicitly or implicitly through BD simulations. The curvature effect is significant in large-sized patches, in the sense that the rate constant of a single disk-like patch on a sphere or a spherical cap largely deviates from the corresponding Hill rate constant, which is obtained when the disk patch is on a plane. Therefore, the theories for N patches based on the Hill rate constant of a single patch cannot be accurate for large-sized patches. However, since the previous comparison was made using BD simulations with small-sized patches and small reactive area fractions, and new theories and methods were recently proposed, it is necessary to extend the comparison to include cases with large-sized patches and new results from recently developed theories and other numerical methods. Specifically, in previous studies, relatively small-sized patches and small reactive area fractions σ were used: a/R = 0.063 with σ≤ 0.25 in the work of Northrup [26]; a/R = 0.025, 0.05, 0.1, and 0.2 with σ≤ 0.5 in the work of Berezkowskii et al. [33]; a/R = 0.063 and 0.314 with σ≤ 0.4 in the work of Wu and Lu [30], where a and R are the radii of a curved disk and sphere; and σ = 0.125 and 0.25 in the work of Lu [29]. In fact, in addition to BD simulations, recently sophisticated numerical methods, such as the spectral boundary element method with Zernike polynomials [36], have been developed for the BP model. While these methods were applied to cases with a very large number of patches (N = 2000 [36] and 100,000 [45]), the values of σ were small; σ≤0.25 in the work of Bernoff and Lindsay [36] and σ = 0.05 in the work of Kaye and Greengrad [45]. However, there has been no study with multiple large-sized patches with a large reactive area fraction. In this work, we investigate various cases, including those with large-sized patches and large total reactive area fractions, using a numerical method called the finite element method (FEM), which has been recently used for the study of a single reactive patch on a sphere [44]. 

Our research goal is fourfold. The first is to investigate whether a symmetric configuration gives the maximum value of the rate constant for the cases of N = 3 and 4, extending the previous study for N = 2. The second is to explore the cases with large-sized patches and large total reactive area fractions of patches using the FEM. The third is to test our recent kinetic theory by comparing its theoretical predictions with the numerical results that were previously reported in the literature (BD simulations) or are newly reported here (FEM). After validating our theory, the last goal is to further investigate the variations of the overall rate constant for a given constraint on patches using our theory.

Accordingly, our paper is organized as follows. In Section 2, we explain the details of the reaction systems and computational methodologies. In Section 3, we study the dependence of patch arrangements on the reaction kinetics for two, three, and four identical patches using the FEM to better understand the nature of competition effects. Then, we further study the reaction kinetics for two, three, four, six, and twelve identical patches with symmetric arrangements for various fixed sizes of patches. The rate constants calculated from the FEM are compared with the rate constants obtained from previous BD simulations and various kinetic theories. Additionally, instead of the fixed sizes of patches, we also study the kinetics of cases where the total reactive area fraction is fixed. After the validation of our theory by comparison with numerical results (FEM and BD simulations), we then use our kinetic theory to discuss the variation of overall rate constants for reactive patches on a sphere. In Section 4, we give our conclusions by summarizing our findings and discussing some implications of our work.

## 2. Methods and Materials

### 2.1. Reaction Models

**Figure 1:**
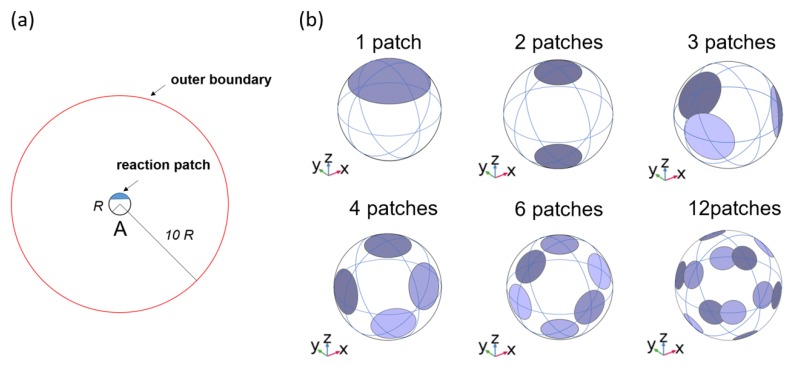
(**a**) Schematic diagram of our chemical reaction model. One reactant molecule A of radius R with a single reactive patch (blue) is located at the center of the coordinate system. An outer boundary condition (red) is located 10 R from the center. (**b**) Equidistant arrangements of N reactive patches on reactant A, with N = 2, 3, 4, 6, and 12, along with a single patch (N = 1), under a fixed fraction of total reactive area over the entire surface area (σ) of 0.2. The bluish spherical caps represent the reactive patches. The figures in (**b**) were prepared by using COMSOL.

In this work, we consider a diffusion-limited (or diffusion-controlled) bimolecular reaction between reactants A and B. For our convenience, we assume that reactant A has a spherical shape with a radius R, has multiple nonoverlapping identical reactive patches on its surface, and is fixed at the origin of the coordinate system. Around reactant A, we assume that there are many point-like molecules of reactant B that do not interact with each other and freely move with a diffusion coefficient D. When a molecule of reactant B contacts one of the reactive patches of reactant A, the reaction occurs immediately. This reaction process can be described by the Smoluchowski equation for the concentration of reactant B. Here, to further simplify, we normalize the concentration of reactant B by the bulk solution concentration of B, which is the concentration far away from A, so that the concentration $c_{B}$ is a dimensionless variable. We also assume that our model system is in a steady state. To solve the Smoluchowski equation, we set up the boundary conditions as follows: (1) on the reactive patches of A, $c_{B}$ is zero (absorbing boundary condition); (2) on the remaining nonreactive surface area of A, the flux of B onto A is zero (reflecting boundary condition); and (3) at locations far away from A, $c_{B}$ is 1 (bulk-solution concentration), meaning no reactions occur when B is far from A. We can define an outer boundary for boundary condition (3); for example, in Figure 1a, the red sphere represents an outer boundary condition. By solving the Smoluchowski equation, we obtain $c_{B}$ and calculate the rate constant k by calculating the fluxes of B at the reactive patches of A. For more details, one can refer to our previous work [44].

In our model, the reactive patches of reactant A have a spherical-cap or disk-like shape, as shown in Figure 1b. For example, in Figure 1b, the disk-like reactive patches, whose fraction of the total reactive area over the entire surface, or σ, is 0.2, are distributed over reactant A. The N patches (N > 1) are symmetrically distributed such that they are maximally distant from each other. In general, these patches can have different sizes and various arrangements, and, moreover, the number of patches can be arbitrary. Here, to simplify, we assume that all the patches are identical. That is, in a given system, all patches are spherical caps or curved disks, and they have the same surface area. For our convenience, we denote the area of a single patch as $\pi a^{2}$, which is the same area as that of a disk with a radius a on a plane, and, here, the length a is regarded as the characteristic length determining the size of a patch. 

Since identical reactive patches on a sphere can have various numbers N and sizes a of patches with various arrangements Ω, the overall rate constant k is a function of N, a, and Ω, or k = k(N, a, Ω). Here, we study the rate constant for the following three cases: (1) (the effect of arrangement) N and a are fixed while Ω is free to change; (2) (the effect of the number of patches) a is fixed while N and Ω are free to change; and (3) (the effect of patch size) the fraction of total reflective area σ is fixed while a, N, and Ω are free to change under a fixed value of σ. In Section 3, we study the detailed kinetics of the systems in each case. 

### 2.2. Computational Methods

**Figure 2:**
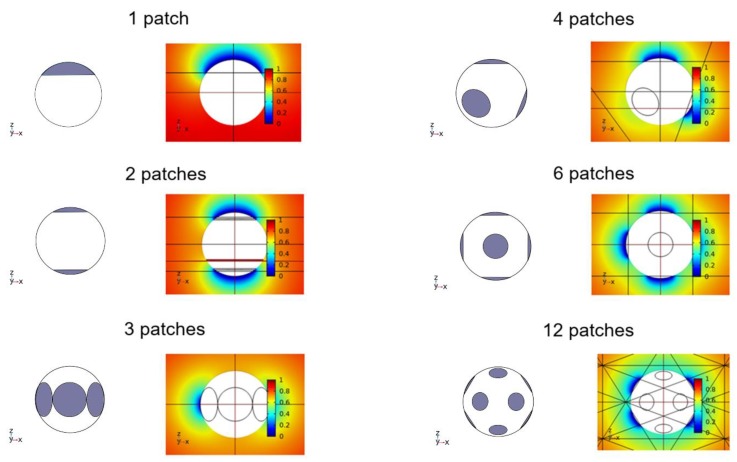
Concentration color maps for symmetric arrangements of N identical reactive patches with σ = 0.2, which are at two opposite poles (N = 2), equilateral triangular (N = 3), tetrahedral (N = 4), octahedral (N = 6), and icosahedral (N = 12), together with the case of a single patch (N = 1). For each case, to clearly show the patch arrangement, we display the patch arrangements without colors in the figures to the left of the color maps. Note that the lines shown here have nothing to do with reactions and are auxiliary lines used in COMSOL. The figures were prepared by using COMSOL.

When σ is 1, or all the surface area of a sphere with a radius R is reactive in an unbounded domain, where the outer boundary is located at an infinite distance from reactant A, the rate constant k is given by $k_{SM}\;=\;4\pi RD$, the Smoluchowski rate constant [1,43]. However, when the surface has nonreactive areas, or σ < 1, the exact closed-form solution of the Smoluchowski equation is not generally known, although an exact but formal expression for the reaction rate was recently derived in a general case, based on a spectral decomposition [46]. Therefore, we need to employ a numerical method to solve the equation, obtain the concentration, and calculate the rate constant. Specifically, in this work, we numerically solve the Smoluchowski equation using the FEM [47,48]. Alternatively, we may use a BD simulation method [26,29,30,33], another numerical method based on a stochastic equation equivalent to the Smoluchowski equation. However, since the FEM allows us to directly solve the Smoluchowski equation even with complicated geometric boundary conditions, we use the FEM. For the FEM calculations, we use the software COMSOL (COMSOL Multiphysics® v.5.4, COMSOL AB, Stockholm, Sweden), an FEM software package. In our systems, to achieve high accuracy in the FEM calculations, we use very fine meshes in the regions of and near the reactive patches; in the mesh construction, the mesh for the region of the reactive patches is “extremely fine” in the predefined setting of COMSOL, or it is finer than the “extremely fine” setting, while the mesh for the remaining regions has “fine” or a more refined mesh setting. Depending on the systems, the numbers of mesh vertices, tetrahedrons, and the element volume ratio (the minimum element volume divided by the maximum element volume) are various; for example, for the systems in Figure 2, the number of mesh vertices is from ~10^5^ to ~10^6^, the number of tetrahedrons is from ~10^6^ to ~10^7^, and the element volume ratio is ~10^−5^ to ~10^−9^. With this mesh setting, calculations with COMSOL give a high accuracy. For example, in reaction models where the exact solutions are known, the relative error between the exact and numerical solutions is less than or approximately 1% [44]. The FEM calculation is typically finished within a few minutes on a standard desktop computer. COMSOL is also used to visualize the results shown in the figures with xmgrace (http://plasma-gate.weizmann.ac.il/Grace/), which is used for plotting and fitting curves to the data.

When we use the FEM, we introduce an outer boundary located 10 R from the center, since it is impossible to create meshes for an infinite space. For a bounded domain that is confined between a spherical reactant with a radius of R at the center and the spherical outer boundary with a radius of 10 R, the exact rate constant k for  σ = 1 is known [44]. This rate constant corresponds to the Smoluchowski rate constant $k_{SM}$ for an unbounded domain with $R_{outer}\;=\;\infty$, and, therefore, it can be denoted ${k^{\prime }}_{SM}$. With this Smoluchowski constant ${k^{\prime }}_{SM}$ for a bounded domain, we define the normalized rate constant $\bar{k}$ in the bounded domain as $\bar{k}\;=\;k/{k^{\prime }}_{SM}$. Additionally, a finding from our previous work [27] indicates that if the size of the outer boundary $R_{outer}$ is larger than the size of patch a, which is satisfied for all our cases, $\bar{k}$ is essentially independent of the value of $R_{outer}$, which means the normalized rate constants are essentially the same whether $R_{outer}$ is 10 R or infinite. That is, $\bar{k}\;=\;\frac{k\;with\;R_{outer}\;=\;10R}{{k^{\prime }}_{SM}\;with\;R_{outer}\;=\;10R}\approx \frac{k\;with\;R_{outer}\;=\;\infty }{k_{SM}\;with\;R_{outer}\;=\;\infty }$. Therefore, we use the FEM results obtained from the bounded systems with $R_{outer}$ = 10 R for comparison with the results from other methods for an unbounded domain, where $R_{outer}$ is at an infinite distance from reactant A. 

As examples of the FEM calculations, Figure 2 shows the solutions of the Smoluchowski equations solved by COMSOL with $R_{outer}$ = 10 R, and these solutions are represented by color maps of the normalized concentration $c_{B}$ around reactant A (sphere at the center). The rate constant k is calculated from the fluxes of reactant B at the reactive patches of reactant A. To calculate the normalization constant used in $\bar{k}$ (1/${k^{\prime }}_{SM}$), we also solve the Smoluchowski equation for the case in which the entire surface is fully reactive, or σ = 1, with $R_{outer}$ = 10 R to obtain ${k^{\prime }}_{SM}$.

### 2.3. Kinetic Theories

**Figure 3:**
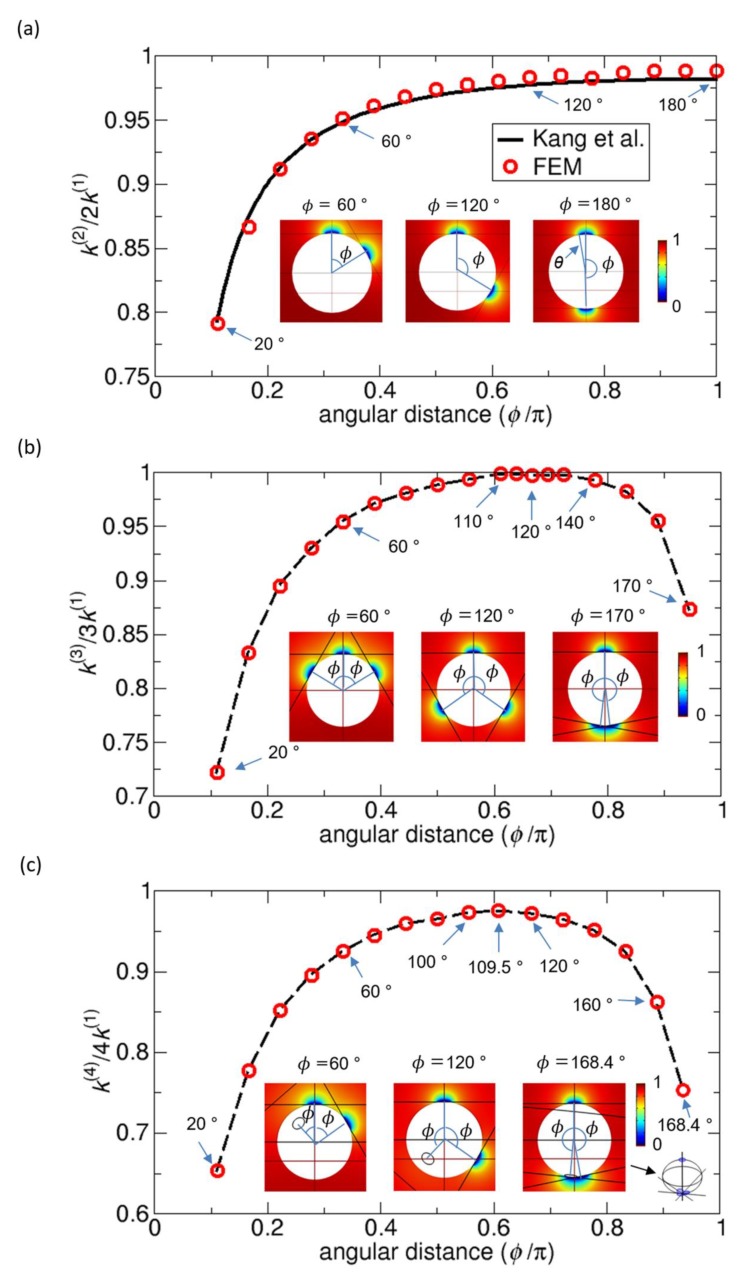
Overall rate constants $k^{\left(N\right)}/\left(Nk^{\left(1\right)}\right)$ for N identical disk-like reactive patches on a sphere (reactant A) as functions of angular distance ø for (**a**) N = 2, (**b**) N = 3, and (**c**) N = 4 from the finite element method (FEM). The rate constants $k^{\left(N\right)}$ are normalized by the rate constants $Nk^{\left(1\right)}$ when the patches are infinitely separated (no competition). For N = 2, we also plot the rate constant calculated from the theory of Kang et al. [13] (solid line). For N = 3 and 4, the dashed lines connecting data points are drawn as a guide for the eye to see the trend. Patches with a polar angle θ = 10° are used to construct the reaction systems, and the area of a single patch is 0.76% of the total surface area. The insets show color maps of the concentration of reactant B around reactant A (white sphere) on a plane passing through the centers of reactant A and the top patch. For N = 2, the relative difference between the rate constants obtained from the FEM and the theory is less than 1%. For N = 3 and 4, the color maps also show the patch arrangements specified by a single parameter of the angle ø, which is the angular distance from the top patch to one of the other patches. In (**c**), note that one patch is not seen in the inset figures, because it is behind the sphere.

As discussed in Section 1, Berg and Purcell first proposed a model (the BP model) for multiple reactive patches on a sphere and formulated the kinetics involved in the model [4]. Specifically, they derived a formula for the rate constant k using a mathematical analogy between chemical kinetics and electrostatics. Their formula for N disk-like reactive patches, each with a patch area of $\pi a^{2}$, on a sphere of radius R is given by

(1) $$k_{BP}\;=\;k_{SM}\frac{Na}{Na+\pi R}$$

where $k_{SM}\;=\;4\pi RD$. Here, the subscript in the rate constant (BP, in this case) is introduced to indicate the theory used to obtain the formula. Additionally, it should be noted that a disk-like patch (spherical cap) whose area is $\pi a^{2}$ is characterized by the polar angle θ (see Figure 3a). From the area equation of $\pi a^{2}\;=\;2\pi R^{2}\left(1-\cos\theta \right)$, we obtain a relation between a and θ, which is $a\;=\;2R\sin\frac{\theta }{2}$.

From Equation (1), the normalized rate constant is defined as ${\bar{k}}_{\;BP}\;=\;\frac{k_{\;BP}}{k_{SM}}$. Furthermore, with $2\sqrt{N}\sqrt{\sigma }\;=\;\frac{Na}{R}$, ${\bar{k}}_{BP}$ can also be expressed in terms of σ($=\;\frac{N\pi a^{2}}{4\pi R^{2}}$), the fraction of the total area of reactive patches over the total surface area of the sphere, as follows: ${\bar{k}}_{\;BP}\;=\;\frac{2\sqrt{N}\sqrt{\sigma }}{2\sqrt{N}\sqrt{\sigma }+\pi }$, which is useful for the cases when σ is fixed.

Shoup and Szabo derived Equation (1) based on the idea that a sphere with N reactive patches can be kinetically equivalent to a sphere with uniformly partial reactivity [31]. Zwanzig further developed this idea of homogenization and proposed an effective medium theory, which gives an improved formula in that it produces the correct value at σ = 1, or it becomes the Smoluchowski constant $k_{SM}$ [32]. Specifically, the Zwanzig (Z) formula for the rate constant is given by

(2) $$k_{z}=k_{SM}\frac{Na}{Na+\pi R\left(1-\sigma \right)}$$

Later, Berezhkovskii et al. combined the BH theory with BD simulation results from random patch arrangements to find a more accurate formula for the rate constant [33,34]. They provided two formulas, but since they produce similar results, we used the later version of the formula [34], which is

(3) $$k_{BH}\;=\;k_{SM}\frac{Na\left(1+0.34\sqrt{\sigma }+0.58\sigma^{2}\right)}{Na\left(1+0.34\sqrt{\sigma }+0.58\sigma^{2}\right)+\pi R{\left(1-\sigma \right)}^{2}}$$

Lindsay, Bernoff, and Ward derived a formula for a uniform distribution of patches using a matched asymptotic expansion analysis, especially for the low surface coverage limit, σ«1, as follows:

(4) $$k_{LBW}\;=\;k_{SM}\frac{Na}{Na+\pi R\left(1-\frac{4}{\pi }\sqrt{\sigma }+\frac{a}{\pi R}\ln\left(4e^{-1}\sqrt{\sigma }\right)+\frac{a^{2}}{2\pi R^{2}\sqrt{\sigma }}\right)}$$

However, this formula gives an incorrect asymptotic value for ${\bar{k}}_{LBW}$, in that $\underset{\sigma \to 1}{\lim}{\bar{k}}_{LBW}\;=\;\frac{2\sqrt{N}}{2\sqrt{N}+\pi \left(1-\frac{4}{\pi }+\frac{2}{\pi \sqrt{N}}\ln\left(4e^{-1}\right)+\frac{2}{\pi N}\right)}$.

In addition to these formulas, we recently derived a new formula [27] based on the surface curvature-dependent kinetic theory [44] with correction for the asymptotic behavior as σ goes to 1 [34]. Our general formula with a parameter ${\bar{\kappa }}_{peak}$ is given by

(5) $$k_{E}\;=\;k_{SM}\frac{Na\left(1+\frac{\pi }{2}A\sqrt{\sigma }-\frac{\pi }{2}B\sigma \right)}{Na\left(1+\frac{\pi }{2}A\sqrt{\sigma }-\frac{\pi }{2}B\sigma \right)+\pi R{\left(1-\sigma \right)}^{2}}$$

where $A\;=\;\frac{2\left(\pi -2\right){\bar{\kappa }}_{peak}}{\pi \left(2{\bar{\kappa }}_{peak}-1\right)}$ and $B\;=\;\frac{\left(\pi -2\right)}{\pi \left(2{\bar{\kappa }}_{peak}-1\right)}$.

From a numerical comparison with an FEM calculation, we determined ${\bar{\kappa }}_{peak}$ in our previous work [27]. Here, we use 0.75 for the value of ${\bar{\kappa }}_{peak}$.

(6) $$k_{E}\;=\;k_{SM}\frac{Na\left(1+\frac{3\left(\pi -2\right)}{2}\sqrt{\sigma }-\left(\pi -2\right)\sigma \right)}{Na\left(1+\frac{3\left(\pi -2\right)}{2}\sqrt{\sigma }-\left(\pi -2\right)\sigma \right)+\pi R{\left(1-\sigma \right)}^{2}}$$

In a way to define ${\bar{k}}_{BP}$, instead of the original forms of rate constants given by (1)-(6), we also use the dimensionless forms of rate constants that are normalized by the Smoluchowski rate constant $k_{SM}$, which are denoted ${\bar{k}}_{Z}$, ${\bar{k}}_{BH}$, ${\bar{k}}_{LBW}$, and ${\bar{k}}_{E}$. We use these formulas in Equations (1)-(6) from various theories for comparison with the numerical results from BD simulations (previous works) and the FEM (our current work), as discussed in Section 3. 

## 3. Results and Discussion

### 3.1. Dependence of Reaction Kinetics on Patch Arrangement

The spatial arrangement of reactants can significantly affect the reaction kinetics due to the competition between the reactants [14]. Likewise, the arrangements of reactive patches on a sphere can affect the overall reaction kinetics through the competitions between patches. To understand the effect of the distribution of reactive patches on the overall rate constant, we first examine the simplest case, two identical patches on a sphere, and see how the overall rate constant is dependent on their relative distances. Here, the distance between the patches on a sphere is characterized by the angular distance ø, the angle formed by the two directional vectors from the center of sphere to the centers of the two patches, as shown in Figure 3a. When ø is zero, the two patches perfectly overlap with each other, and when ø is π, they are located at opposite poles of the sphere. For this study, we use two patches with a polar angle θ = 10°, which are shown in Figure 3a. Therefore, the contact angle ø between the two patches is $\frac{20\pi }{180}$ or 20°. By using the FEM, we calculate the overall normalized rate constant defined as $k^{\left(2\right)}/2k^{\left(1\right)}$, a function of ø for a range from $\frac{20\pi }{180}$ to π, or from 20° to 180°. Here, the overall rate constant for two patches on a sphere, or $k^{\left(2\right)}$, is normalized by the rate constant when the two patches are independent of each other (no competition), or $2k^{\left(1\right)}$, which is two times the rate constant of a single patch on a sphere, or $k^{\left(1\right)}$. The result obtained from the FEM calculation is shown in Figure 3a.

In Figure 3a, to validate our numerical FEM results, we compare the FEM results with the results from the theory by Kang et al. [13]. They show good agreement in that the relative difference is less than 1%. The plots show the trend that, as ø increases, the overall normalized rate constant increases. From this trend, one may expect that the maximum would be at ø = 180°. In fact, the maximum value of the normalized rate constant appears at *ø* = 170°. However, considering the numerical error, two values at ø = 170° and 180° are essentially the same, and we may say that the expectation is true. 

From the results in Figure 3a, we can address three points. First, the normalized rate constant can be very close to 1 but is less than 1. This means that because of the competition between the patches on a sphere, the rate constant of each patch is reduced, compared to the rate constant in the absence of competition. Second, the competition effect observed here is short-ranged in the sense that, when two patches are separated by larger than the angular distance of three times their own size, which is ø = 60°, the normalized rate constant is larger than 0.95. Third, the normalized rate constant is very close to 1 near ø = 180°, which implies that, when the patches are maximally separated, the competition almost disappears. From this analysis, we may conclude that, in general, as the angular distance ø increases or the two patches become more separated, the overall rate constant increases. Therefore, to maximize the overall rate constant for two identical patches, we need to arrange them at two opposite poles, which means the patch distribution has a $D_{\infty h}$ symmetry, as in homonuclear diatomic molecules. 

Furthermore, we study the cases of three and four identical reactive patches with θ = 10° that are the same patches used in the two-patch case shown in Figure 3a. To generate various patch distributions, we consider the change in the angular distance ø from the top patch to one of the other patches, which is defined as the angle between the directional vector from the center of the sphere to the center of the top patch and the directional vector to the center of any of the other patches, as illustrated in Figure 3b,c. Note that, in this variation scheme, the angular distances *ø* are the same, and, moreover, in the four-patch case, the three patches except for the top patch are equally distributed on a plane passing through their centers. Therefore, once a single parameter of ø is determined, the relative arrangement of patches is completely determined. However, for a more general case, one may consider the cases where the patches are arbitrarily arranged. Note that when ø = 120° for three patches and ø = 109.5° for four patches, the patch arrangements are most symmetric and are equilateral triangular and tetrahedral, respectively. 

Figure 3b,c show the rate constants as functions of ø for three and four patches, which are also normalized by the rate constants $3k^{\left(1\right)}$ and $4k^{\left(1\right)}$, respectively, when the reactive patches do not compete with each other. For three patches, within numerical errors, the maximum value of the normalized rate constant is at ø = 120°. Likewise, for four patches, the maximum value is at ø = 109.5°. Note that, when θ = 168.4°, the three patches, except for the top patch, are aggregated near the opposite side to the top patch, and they contact each other without overlaps, as shown in the inset of Figure 3c. 

From these considerations, in general, to obtain the maximum rate constants, one may need to arrange the patches in symmetric ways, such that the reactive patches are maximally separated, as in the Thompson (or VSEPR) or Tammes problems. The reason for this is that, as the patches become more separated, the competition between them is reduced, and, consequently, the overall rate constant is higher. 

**Figure 4:**
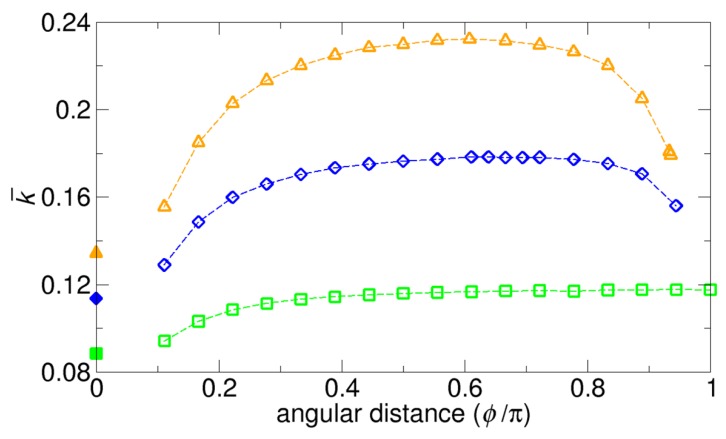
Rate constants $\bar{k}$ normalized by the Smoluchowski rate constant $k_{SM}$ for the systems in Figure 3 with two (green square), three (blue diamond), and four (orange triangle) reactive patches on a sphere. Note that the differences between Figure 3 and this figure comes from different normalizations. The values at ϕ = 0° correspond to the rate constants calculated when the patches are merged into single patches that have the same total reactive area as the patches before merging.

Instead of normalizing the overall rate constant $k^{\left(N\right)}$ for N patches by the rate constants $Nk^{\left(1\right)}$ in the absence of competitions, we can also use the Smoluchowski rate constant $k_{SM}$ for the normalization, so that we can compare the three rate constants in one plot, which is shown in Figure 4. This normalized rate constant can be denoted $\bar{k}$, and this normalization is a typical way to represent the rate constants in dimensionless form. The plots in Figure 4 indicate that, for a given size of a patch (in this case, θ = 10°), as the number of reactive patches increases from N = 2 to N = 4, the rate constant $\bar{k}$ increases accordingly because of the increase in the total reactive area (see any vertical lines parallel to the y-axis). On the other hand, for a given number of patches, the rate constant has a range of values, depending on the arrangement of the patches (see the variation along each colored curve). That is, the rate constant $\bar{k}$ is the minimum when the patches are aggregated without overlaps or the competition effect is maximized, and it is the maximum when the patches are arranged in the most symmetric way or the competition effect is minimized. Moreover, if we assume that the patches are merged into one larger single patch while keeping the total reactive area fixed, we have a lower value of the minimum, as is represented by the solid mark on the y-axis in Figure 4. In this case, the competition effect is stronger. In addition to the variation above under the fixed number of patches, the variations in the rate constant under other types of constraints are also discussed in the following sections in detail.

Additionally, the maximum values of $\bar{k}$ in Figure 4 can be used to evaluate the accuracy of the various kinetic theories, since the maximum values are also calculated from the formulas given by Equations (1), (2), (3), (4), and (6), with $\bar{k}\;=\;\frac{k}{k_{SM}}$ and $a\;=\;2R\sin\frac{\theta }{2}$. A comparison between the FEM results and the results from the kinetic theories is given in Table 1. Our theory and the LBW theory give the closest values to those from the FEM numerical results.

### 3.2. Dependence of Reaction Kinetics on the Number of Reactive Patches Under a Fixed Patch Size

In Section 3.1, we discuss the effect of patch arrangement on the overall rate constant. However, instead of the patches being arranged in specific ways in Section 3.1, the patches would be randomly distributed on a sphere. In this case, we can raise a fundamental question: what is the average overall rate constant for random patch arrangements? 

Interestingly, from BD simulations, Northrup found that the ensemble-averaged value of the rate constant for random patch arrangements is very close to the value of the rate constant for the symmetric arrangement with maximum patch separation [26]. In fact, from an analysis of the distribution of rate constants for an ensemble, Northrup discussed that, in a large number of arrangements, the patches are located at large distances from one another and behave almost independently, and thus, the distribution of rate constants is biased toward the maximum value of the rate constant, at which the competition effect is minimized [26]. Berg and Purcell also discussed that the diffusive current (flux) to reactive patches for a random patch arrangement would be only slightly smaller than that for the uniform (symmetric) patch arrangement if the number of patches is sufficiently large [4]. Later, Wu and Lu also investigated the distributions of rate constants in BD simulations of random patch arrangements with two fixed patch sizes, and they found that the distributions of rate constants are very narrow for both cases [30], which is consistent with Northrup’s results [26]. 

The reasons why the distribution of rate constants is narrow and the average value of the rate constant is close to the maximum value may be understood from an analogy of the spatial distribution of N particles in a box. That is, when we randomly place N particles in a box, the most probable configuration is that the particles are homogeneously or evenly distributed in the box. Therefore, if we consider the probability distribution of N particle configurations, the probability distribution has the maximum probability when the particles are evenly distributed. Furthermore, if N increases, we expect that the probability distribution of the configurations will become narrower around the maximum probability. Likewise, for the distribution of patch arrangements, the most probable patch arrangement is that the patches are evenly distributed, which we call the symmetric patch arrangement. Therefore, because of this characteristic of the probability distribution, the ensemble-averaged value of the rate constant for the random patch arrangements is close to the maximum value of the rate constant corresponding to the symmetric patch arrangement. 

From the above discussion, once the number of patches, N, and the size of patches, a or θ, are given, the ensemble-averaged rate constant for random patch arrangements is practically completely determined from the most symmetric arrangement, without consideration of all possible patch arrangements. Therefore, in k = k(N, a, Ω), when we consider only an average value of the rate constant, the variation in patch arrangement Ω does not play an important role, and the dependence of the overall rate constant k on N and a (or θ) is more interesting.

To study the effect of the number of patches N on the overall normalized rate constant $\bar{k}$, we can again consider the BP model. To study with this BP model, Northrup considered multiple reactive patches with a patch size of θ =π/50 = 3.6° on a sphere and performed a series of BD simulations with different numbers of patches randomly distributed on a sphere. Northrup also compared the BD simulation results with results from the BP theory and concluded that the BP results only deviated to some extent from the BD simulation results in the intermediate patch coverage regime. However, this comparison was conducted with only small-sized patches whose surface area fraction was 0.000987, or 0.0987%, and up to N = 256, which corresponds to a σ value of 0.253, or 25.3%. Therefore, to completely understand the kinetics, it is necessary to extend this study up to the full coverage (σ~1) and to consider cases with larger patches, where the curvature effect is not ignorable. Moreover, since this comparison was made by Northrup, there has been substantial progress in theoretical approaches and numerical methods, and, thus, we need to include results obtained from other new theoretical and numerical methods in the comparison. In the following subsections, we first consider the original Northrup reaction system with an extended comparison of the rate constant, and then we consider the same Northrup reaction system but with larger sizes of patches. We also use these results to evaluate the accuracy of the theories addressed in Section 2.3.

#### 3.2.1. Original Northrup Reaction System

First, we focus on the original reaction system studied by Northrup [26]. In the Northrup system, the size of a reactive patch is specified by the angle θ = 3.6° (or $\frac{\pi }{50}$). This patch size corresponds to a surface area fraction of 0.000987, which means that we can put ~1000 patches on a sphere without overlap between patches. Therefore, we consider N = 1000 (σ = 0.987) as an upper limit of N in this study. Additionally, we extend the original comparison by Northrup by adding the results from another numerical method, the FEM, and other theories. In fact, we performed this comparison without the FEM and LBW results up to N = 256 in our previous work [27]. 

For the comparison with the theories, we use the formulas in Equation (1) for the BP theory, Equation (2) for the Zwanzig (Z) theory, Equation (3) for the BH theory, Equation (4) for the LBW theory, and Equation (6) for our theory with $\bar{k}\;=\;\frac{k}{k_{SM}}$, $a\;=\;2R\sin\frac{\theta }{2}$, and $\sqrt{\sigma }\;=\;\sqrt{N}\sin\frac{\theta }{2}$. Additionally, for a comparison with a single patch into which all the patches are merged under the same value of σ, we also add the rate constant for the single patch, which corresponds to the lower bound of the rate constant for a given σ value. To calculate the rate constant for a single patch, we use the formula obtained from our curvature-dependent kinetic theory. Specifically, the formula for the rate constant normalized by the Smoluchowski rate constant $k_{SM}$ is given by

(7) $${\bar{k}}_{E,single}\left(\sigma \right)\;=\;-\frac{2\left(\pi -2\right)}{\pi }\sigma^{3/2}+\frac{3\left(\pi -2\right)}{\pi }\sigma +\frac{2}{\pi }\sigma^{1/2}$$

where $\sigma \;=\;\frac{N\pi a^{2}}{4\pi R^{2}}$ [27]. The accuracy of this formula is discussed in Appendix A. 

**Figure 5:**
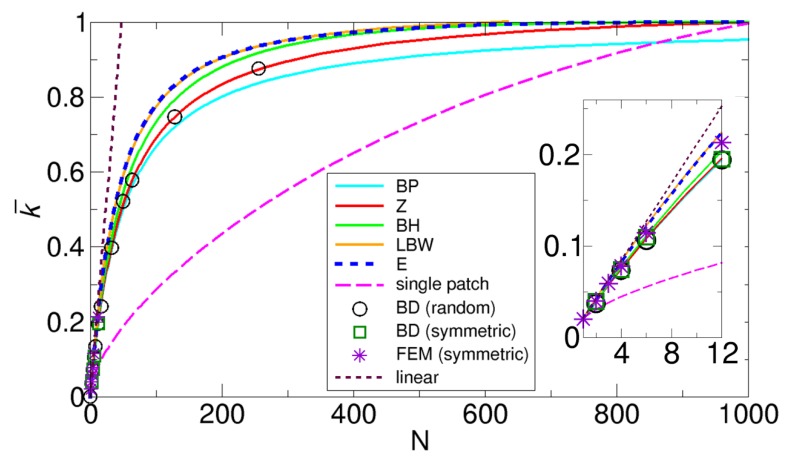
Comparison of the normalized rate constants $\bar{k}$ as functions of the number of patches N, obtained from various methods for the Northrup system with a patch size of θ = 3.6° up to N = 1000 (σ~1). The normalized constants are calculated from the Berg-Purcell (BP), Zwanzig (Z), boundary homogenization (BH), Lindsay-Bernoff-Ward (LBW), and our (E) theory, whose formulas for $\bar{k}$ are obtained from Equations (1), (2), (3), (4), and (6), respectively, and the numerical methods from BD simulations, whose results were reported in the Northrup work [26], and the FEM. We also plot the rate constant when all the patches are merged into one larger single patch (single patch) and when there is no competition, so that the overall rate constant is simply linear with the number of patches N (linear). The inset provides a zoomed-in view for a range of N up to 12 for a detailed comparison. Note that, in the inset, the ensemble-averaged rate constants from the BD simulations of random arrangements (black circles) are very close to the rate constants from the BD simulations of symmetric arrangements (green squares). Additionally, note that the lines from the LBW and our theory are very close to each other.

An extended comparison is shown in Figure 5. As discussed in our previous work [27], the comparison indicates that the Zwanzig results give the best agreement with the Northrup simulation results among the theories. However, as shown in the inset of Figure 5, compared to our FEM results, the normalized rate constant from the Zwanzig theory is underestimated, and the LBW theory, our theory, and the BH theory give better agreement with the FEM results than the Zwanzig theory. 

**Figure 6:**
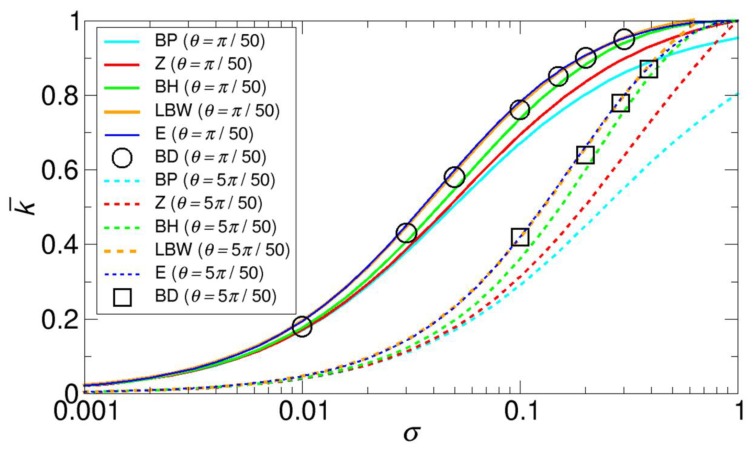
Comparison of the normalized rate constants $\bar{k}$ as functions of the total reactive area fraction σ, obtained from various methods with that from the BD simulations performed by Wu and Lu [30], with patch sizes of θ = 3.6° ($\frac{\pi }{50}$) and 18° ($\frac{5\pi }{50}$). Note that the x-axis is the logarithmic scale. The normalized rate constants for the Berg-Purcell (BP), the Zwanzig (Z), the boundary homogenization (BH), the Lindsay-Bernoff-Ward (LBW), and our (E) theory are calculated from Equations (1), (2), (3), (4), and (6), respectively. Note that the lines from the LBW and our theory are very close to each other.

Additionally, since another set of BD simulation results with θ = $\frac{\pi }{50}$ and $\frac{5\pi }{50}$ is available from the Wu and Lu work [30], we also compare the normalized rate constants $\bar{k}$ from the BD simulations and various theories in terms of the total reactive area fraction σ. For direct comparison with the original data provided by Wu and Lu, we use σ in the comparison, but we can easily convert from σ to N through $\sigma \;=\;\frac{N\pi a^{2}}{4\pi R^{2}}\;=\;N{\left(\sin\frac{\theta }{2}\right)}^{2}$ The results are displayed in Figure 6. A comparison with the results from the BD simulations clearly shows that the LBW theory, our theory, and the BH theory give the best agreement with the BD simulation results, while the Zwanzig theory only gives the best agreement with the Northrup simulation results shown in Figure 5. These differences may be due to differences in the details of the BD simulation methods.

Here, we can point out two features observed in Figure 5 and Figure 6. One feature is that as N increases, $\bar{k}$ from all the theories except for the BP and the LBW theories correctly approaches 1. For example, as shown in Figure 5, $\bar{k}$ from the BP and the LBW theories approach $\frac{2\sqrt{N_{max}}}{2\sqrt{N_{max}}+\pi }\approx 0.953$ and $\frac{2\sqrt{N_{max}}}{2\sqrt{N_{max}}+\pi \left(1-\frac{4}{\pi }+\frac{2}{\pi \sqrt{N_{max}}}\ln\left(4e^{-1}\right)+\frac{2}{\pi N_{max}}\right)}\approx 1.013$, respectively, where $N_{max}\;=\;\frac{1}{\;0.000987\;}\;=\;1013$ as σ→1. Another feature shown in Figure 5 is that the normalized rate constant for a single patch is smaller than that for multiple patches for the same reactive area fraction, and this feature means that, when reactive patches are dispersed rather than aggregated, the rate constant is larger. Consistent with this feature, the last feature from Figure 6 is that for a given σ value, small-sized patches with $\theta \;=\;\frac{\pi }{50}$ have a larger rate constant than large-sized patches with $\theta \;=\;\frac{5\pi }{50}$. This feature is related to the reduction in competitions between patches. We discuss this size effect in detail in Section 3.3.

#### 3.2.2. Northrup Reaction System with Larger Patches

**Figure 7:**
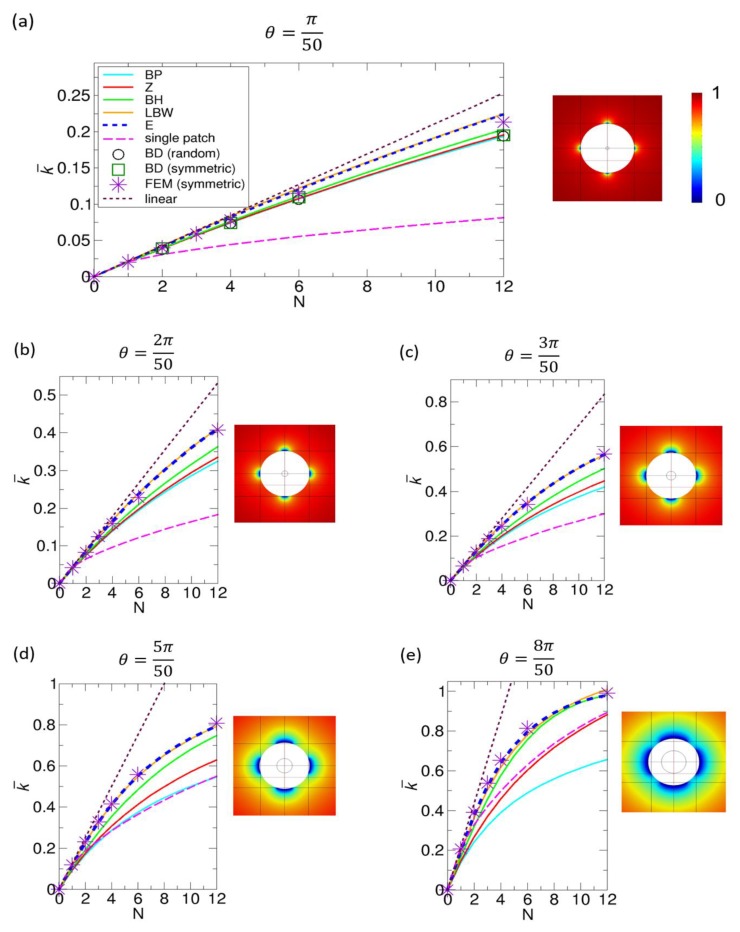
Comparison of the normalized rate constants $\bar{k}$ as functions of the number of patches N, obtained from various methods for various sizes of reactive patches, whose θ are (**a**) 3.6° (π/50), (**b**) 7.2° (2π/50), (**c**) 10.8° (3π/50), (**d**) 18° (5π/50), and (**e**) 28.8° (8π/50) for a range of N up to 12. The reactive area fraction of a single patch for each case is 0.000987 for π/50, 0.00394 for 2π/50, 0.00886 for 3π/50, 0.0245 for 5π/50, and 0.0618 for 8π/50. The figures next to the plots show the concentration color maps for six patches. Since the maps are on two-dimensional planes, only four patches are shown.

To better understand the cases of large-sized patches, for which the curvature effect cannot be negligible, we also consider the Northrup reaction systems with larger patch sizes of $\theta \;=\;\frac{2\pi }{50},\frac{3\pi }{50},\frac{5\pi }{50}$, and $\frac{8\pi }{50}$. The results, including the original Northrup patch with $\theta \;=\;\frac{\pi }{50}$, are displayed in Figure 7. Remarkably, for larger patches, the results from the LBW and our kinetic theory give the best agreement with the FEM numerical results. The BH theory also gives reasonably good agreement with the FEM results, while the other theories do not. This is because the BH theory implicitly takes into account the curvature effect by incorporating BD simulation results into the theory. Specifically, the other theories are based on the assumption that a patch on a sphere is so small that it can be considered as a disk on a plane, which does not hold for larger patches.

To illustrate why the curvature effect should be considered in a large-sized patch, we compare the normalized Hill rate constant for a planar disk and the normalized rate constant for a curved disk (or spherical cap) on a sphere, as given by Equation (7). Here, note that both disks have the same area of $\pi a^{2}$. Since the Hill rate constant is 4aD, the corresponding normalized rate constant ${\bar{k}}_{Hill}$ is given by ${\bar{k}}_{Hill}\;=\;\frac{4aD}{4\pi RD}\;=\;\frac{2}{\pi }\sin\left(\frac{\theta }{2}\right)$, where $a\;=\;2R\sin\frac{\theta }{2}$ is used. For a single patch, since $\sqrt{\sigma }\;=\;\sin\frac{\theta }{2}$, we can rewrite ${\bar{k}}_{Hill}$ as follows: ${\bar{k}}_{Hill}\;=\;\frac{2}{\pi }\sigma^{1/2}$, which is the last term of ${\bar{k}}_{E,single}$ in Equation (7). Therefore, if we use the Hill rate constant for a curved disk, we ignore the first two terms in Equation (7). For a small patch, these two terms may be ignorable, but, for a large patch, the two terms should be taken into account. Specifically, when θ is $\frac{\pi }{50}$ for the Northrup patch, σ = 0.000987, and, accordingly, ${\bar{k}}_{Hill}$ = 0.0200 and ${\bar{k}}_{E,single}$ = 0.0210. Therefore, the relative difference, or $\left({\bar{k}}_{E,single}-{\bar{k}}_{Hill}\right)/{\bar{k}}_{E,single}$, in percentage is only ~5%. However, as the patch size increases with $\theta \;=\;\frac{2\pi }{50},\frac{3\pi }{50},\;\frac{5\pi }{50}$, and $\frac{8\pi }{50}$, the relative differences between ${\bar{k}}_{Hill}$ and ${\bar{k}}_{E,single}$ in percentage increase by 9%, 13%, 19%, and 26%, respectively. This means that, for a curved disk, the Hill constant is underestimated compared to the more accurate rate constant from our theory. Physically, the Hill constant does not take into account the flux from below the plane on which a disk is placed, and this limitation explains why the Hill constant is underestimated for a curved disk. Furthermore, in a limiting case with θ = π (or σ = 1), ${\bar{k}}_{Hill}\;=\;2/\pi$ and ${\bar{k}}_{single}\;=\;1$. Consequently, the relative difference is 36%, which means that there should be errors for large patches if we use the theories based on the Hill constant. 

### 3.3. Dependence of Reaction Kinetics on the Number of Reactive Patches Under a Fixed Total Reactive Area Fraction

In addition to the number of patches N, another patch parameter that we may control for reaction kinetics is the total reactive area fraction σ. In this case, under a fixed value of σ, one variable that we can change is the size of a patch, which is characterized by a in area $\pi a^{2}$. Here, note again that, for identical patches in our cases, once a is determined, N is accordingly determined to have a fixed value of σ, where $N\;=\;\frac{4R^{2}}{a^{2}}\sigma$. In this work, we study the dependence of the overall rate constant on the number of reactive patches, as in the previous study by Lu [29], although a study of the dependence on the size of patches is equivalently possible.

#### 3.3.1. Original Lu Reaction System

**Figure 8:**
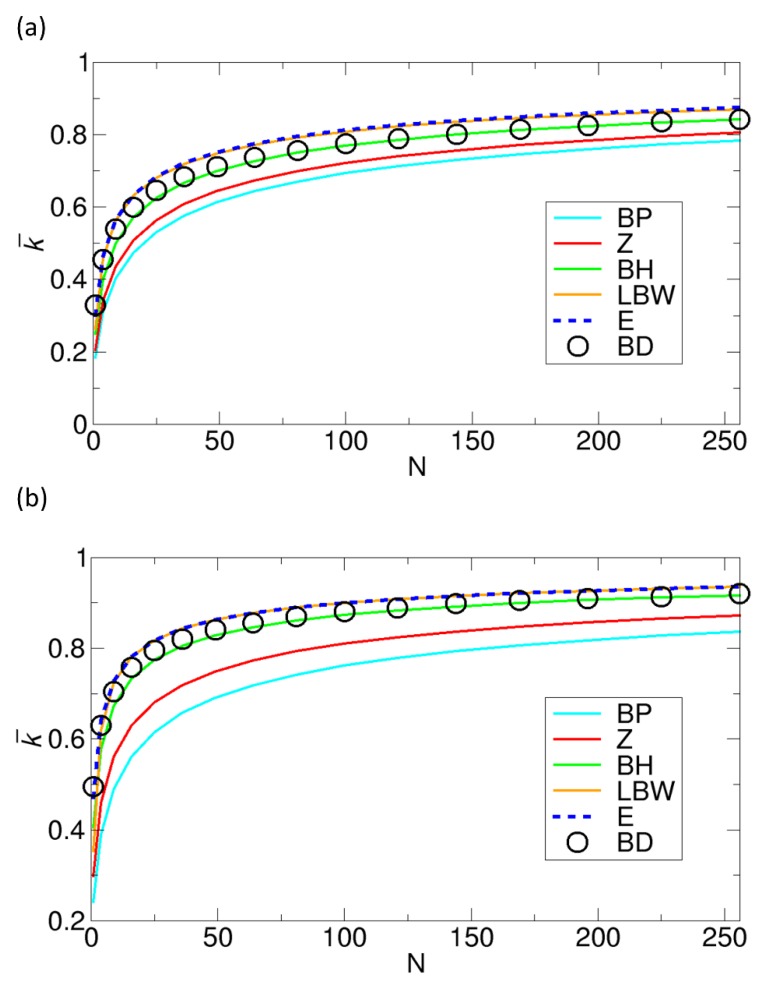
Comparison of the normalized rate constants $\bar{k}$ as functions of the number of patches N, obtained from various theories with the rate constant from BD simulations with random patch arrangements by Lu [29] under fixed values of σ of (**a**) 0.125 and (**b**) 0.25. The normalized rate constants for the Berg-Purcell (BP), the Zwanzig (Z), the boundary homogenization (BH), the Lindsay-Bernoff-Ward (LBW), and our (E) theory are calculated from Equations (1), (2), (3), (4), and (6), respectively.

Lu studied the effect of patch size under fixed values of σ by calculating the rate constant $\bar{k}$ as a function of N from BD simulations of random patch arrangements [29]. Here, we calculate the rate constants from the various theories to compare with that from Lu’s BD simulation results. This comparison is shown in Figure 8. For this comparison, we use Equation (1) (BP theory), Equation (2) (Zwanzig theory), Equation (3) (BH theory), Equation (4) (LBW theory), and Equation (6) (our theory). In this comparison, the BH theory gives the best agreement with the BD simulation results, and the LBW and our theory also show reasonably good agreement, especially for low values of N. However, in the comparison, the BD simulations were performed under relatively small values of σ, 0.125 and 0.25, which means that each patch size is limited by these values of σ. Therefore, we also study cases with higher values of σ in the following section, where we can have larger patches.

#### 3.3.2. Lu Reaction System with Larger Fixed Reactive Area Fractions

**Figure 9:**
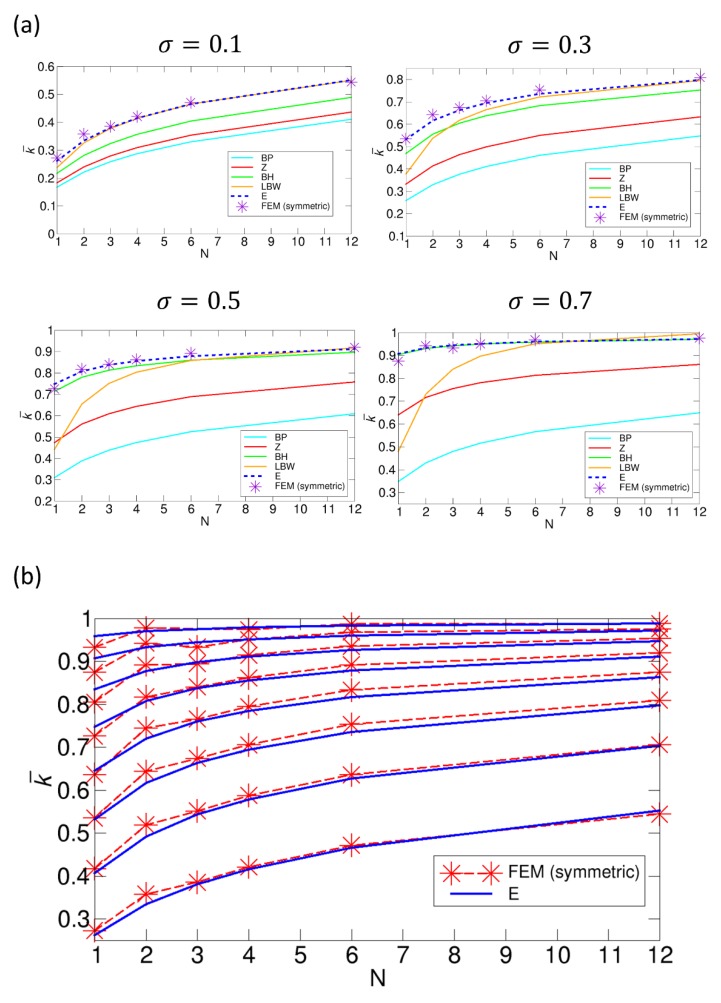
(**a**) Comparison of the normalized rate constants $\bar{k}$ as functions of the number of patches N, obtained from various methods under fixed σ values of 0.1, 0.3, 0.5, and 0.7. The normalized rate constants for the Berg-Purcell (BP), the Zwanzig (Z), the boundary homogenization (BH), the Lindsay-Bernoff-Ward (LBW), and our (E) theory are calculated from Equations (1), (2), (3), (4), and (6), respectively. (**b**) Comparison of the results from our theory (E) with the FEM results for symmetric patch distributions (FEM) from σ = 0.1 (bottom) to σ = 0.8 (top) with an increment of 0.1.

To investigate cases including those with large patches, we examine the reaction systems with fixed σ values of 0.1, 0.3, 0.5, and 0.7 using the FEM numerical method for various symmetric patch arrangements, as is shown in Figure 9a. In particular, we focus on the number of patches up to 12. This is because, beyond 12 patches, it is much more difficult to construct meshes with symmetric (or even) patch arrangements in the FEM. Specifically, for the symmetric patch arrangement for a large value of N, we may use Fibonacci spiral points, which are approximately uniformly distributed on a sphere [35,36], but, as N increases, it is more difficult to successfully generate the fine meshes compatible with N spherical-cap boundaries on the sphere. However, other methods, such as the Brownian dynamics simulation method [26,29,30] and matched asymptotic analysis [35,36], can handle the systems with a much larger number of N, as shown in Figure 5, Figure 6, and Figure 8. 

Additionally, the FEM results are also used as a testbed for testing the accuracy of the formulas provided by the various theories. In fact, once we verify the accuracy of our theory, we use it for an extended study, which is discussed in Section 3.4.

In Figure 9a, we compare the rate constants from various theories with the rate constant from the FEM results. This comparison shows that our result and the FEM results are in excellent agreement, compared with the results from the other theories. It should be noted that the rate constant from the LBW theory is largely deviated from the one from the FEM calculations as σ increases and N decreases. This is because, for a single patch, the accuracy of the LBW formula given by Equation (4) is lower as the curvature of the patch increases, as shown in Appendix A. From the comparison in Figure 9a, we may conclude that our formula for the rate constant can be used for the full range of σ with reasonable accuracy. To test this claim, we perform an extended comparison with our theory in Figure 9b. That is, using our theory, we calculate the rate constant from σ = 0.1 to σ = 0.8 with an increment of 0.1 and compare the theoretical results with the FEM results, and this comparison also shows good agreement. In addition, interestingly, we notice that, for σ = 0.7, the rate constant for three patches has a lower value than that for two patches. This indicates that it is not necessarily true that an arrangement with a larger number of smaller patches always gives a higher rate constant in symmetric arrangements. This is because, when σ is high, three patches at equilateral triangular positions are overall closer to each other than two patches at linear positions (north and south poles), and, thus, the former has a stronger competition effect, which further reduces the rate constant. On account of this closeness among three patches at equilateral triangular positions, at σ = 0.8, the three patches overlap with each other, which explains why the point for N = 3 at σ = 0.8 is missing in the plot shown in Figure 9b. Additionally, note that the case of σ = 0.9 is not calculated, because, for all cases except for N = 1 and 2, the patches overlap in the symmetric arrangements.

### 3.4. Variation in the Overall Rate Constant

One interesting quantity is the variation in the rate constant, which can be defined by the difference between the upper and lower bounds of the normalized rate constant. As we discuss in Section 3.1, for a given number of patches N under a fixed size a of patches, we show that the rate constant has various values due to the patch arrangement. That is, when the patches are aggregated or maximally separated, the rate constant has the minimum or maximum value, respectively. We can estimate this variation in the rate constant using our theory. This is because, from the comparisons with the numerical calculations of the FEM (large patches) and BD simulations (small patches) in Section 3.1, Section 3.2, and Section 3.3, we find that the formulas of our theory can be used to calculate the rate constant for any cases of patches with reasonably high accuracy. Specifically, to obtain the maximum and minimum values, we can use our formulas for the rate constant given by Equation (6). The minimum value, or ${\bar{k}}_{min}$, is calculated using Equation (6) with N = 1 for a single patch, and the maximum value, or ${\bar{k}}_{max}$, is obtained using Equation (6) with N for the symmetric patch arrangement. Therefore, for the given N and a, the range of the normalized rate constant $\bar{k}$ is given by the following inequality:

(8) $$\frac{2\sqrt{\sigma }\left(1+\frac{3\left(\pi -2\right)}{2}\sqrt{\sigma }-\left(\pi -2\right)\sigma \right)}{2\sqrt{\sigma }\left(1+\frac{3\left(\pi -2\right)}{2}\sqrt{\sigma }-\left(\pi -2\right)\sigma \right)+\pi {\left(1-\sigma \right)}^{2}}\leq \bar{k}\;\;\leq \frac{Na\left(1+\frac{3\left(\pi -2\right)}{2}\sqrt{\sigma }-\left(\pi -2\right)\sigma \right)}{Na\left(1+\frac{3\left(\pi -2\right)}{2}\sqrt{\sigma }-\left(\pi -2\right)\sigma \right)+\pi R{\left(1-\sigma \right)}^{2}}$$

Note again that σ is the total reactive area fraction, which is given by $\frac{Na^{2}}{4R^{2}}$. It also should be noted that this inequality is not mathematically strict but approximate, because the upper and lower bounds of $\bar{k}$ are estimated by our approximate solution of ${\bar{k}}_{E}$.

Accordingly, the variation $∆\bar{k}$ is given by the difference between the maximum and minimum values, which is

(9) $$\begin{array}{cc} ∆\bar{k}\;=\;{\bar{k}}_{max}-\;{\bar{k}}_{min}\; & \\ & =\;\left(\frac{Na\left(1+\frac{3\left(\pi -2\right)}{2}\sqrt{\sigma }-\left(\pi -2\right)\sigma \right)}{Na\left(1+\frac{3\left(\pi -2\right)}{2}\sqrt{\sigma }-\left(\pi -2\right)\sigma \right)+\pi R{\left(1-\sigma \right)}^{2}}\right)\\ & -\left(\frac{2\sqrt{\sigma }\left(1+\frac{3\left(\pi -2\right)}{2}\sqrt{\sigma }-\left(\pi -2\right)\sigma \right)}{2\sqrt{\sigma }\left(1+\frac{3\left(\pi -2\right)}{2}\sqrt{\sigma }-\left(\pi -2\right)\sigma \right)+\pi {\left(1-\sigma \right)}^{2}}\right) \end{array}$$

Note that for N = 1, $∆\bar{k}$ = 0.

It also should be noted that in estimating the lower bound, or the rate constant for a single patch, we can use a more accurate formula given by Equation (7) for ${\bar{k}}_{E,single}\left(\sigma \right)$. However, the difference between the two lower bounds by Equations (6) and (7) is not significant (see Appendix A). Therefore, we use Equation (9) for estimating the variation $∆\bar{k}$. The calculation of $∆\bar{k}$ using Equation (9) can be immediately applicable to the cases in Section 3.2 and Section 3.3.

#### 3.4.1. Variation in the Overall Rate Constant in the Northrup Reaction Systems

**Figure 10:**
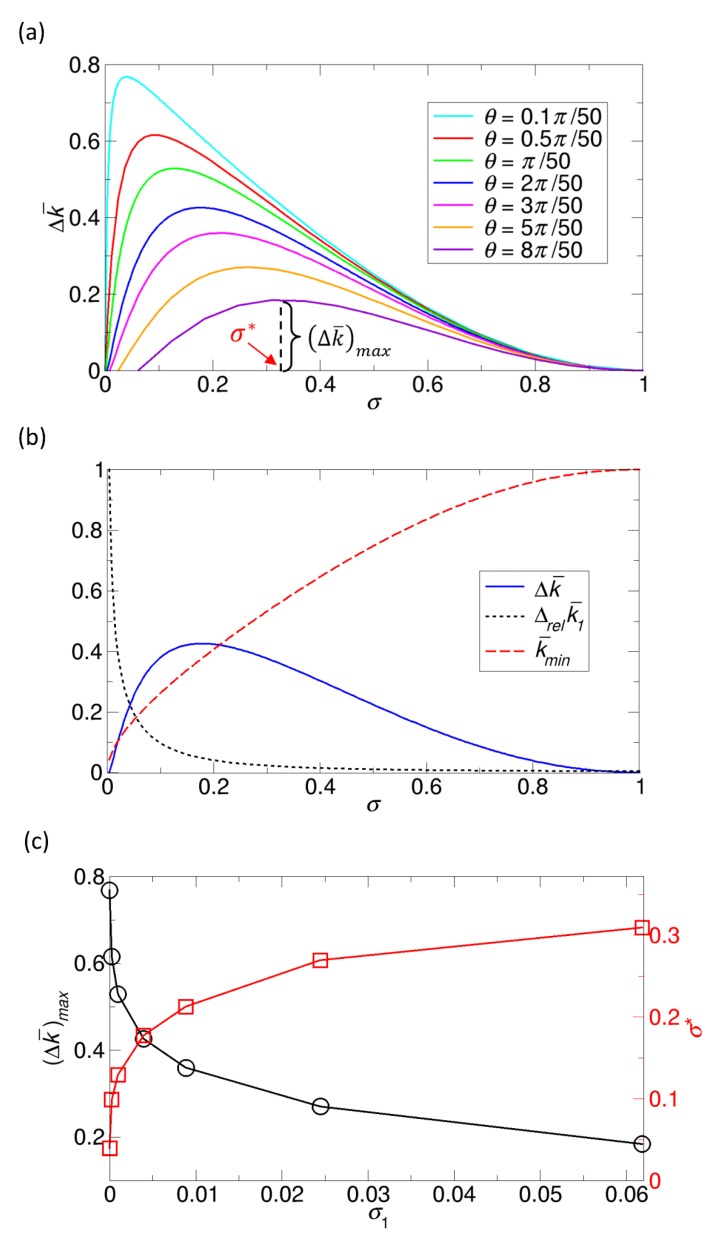
(**a**) Variation $∆\bar{k}$ calculated from Equation (9) for the Northrup reaction systems as a function of the total reactive area fraction σ for patch sizes of $\theta \;=\;\frac{0.1\pi }{50},\frac{0.5\pi }{50},\frac{\pi }{50},\frac{2\pi }{50},\frac{3\pi }{50},\frac{5\pi }{50}$, and $\frac{8\pi }{50}$. The patches with $\theta \;=\;\frac{0.1\pi }{50},\frac{0.5\pi }{50},\frac{\pi }{50}\;,\frac{2\pi }{50}\;,\frac{3\pi }{50}\;,\frac{5\pi }{50}$, and $\frac{8\pi }{50}$ have reactive area fractions ($\sigma_{1}$) of 9.87×10^−6^, 0.000247, 0.000987, 0.00394, 0.00886, 0.0245, and 0.0618, respectively. The sigma value ($\sigma^{*}$) giving the maximum value of variation ${\left(∆\bar{k}\right)}_{max}$ is marked for $\theta \;=\;\frac{8\pi }{50}$ in the plot. (**b**) $∆\bar{k}$, ${∆}_{rel}{\bar{k}}_{1}\left(\sigma \right)$, and ${\bar{k}}_{min}$ as functions of σ for the case of $\theta \;=\;\frac{2\pi }{50}$. (**c**) ${\left(∆\bar{k}\right)}_{max}$ (black) and the corresponding $\sigma^{*}$ (red) as functions of the reactive area fraction of a single patch ($\sigma_{1}$).

In Section 3.2, we consider the Northrup cases with fixed patch sizes specified by a or, equivalently, θ. For those cases with $\theta \;=\;\frac{0.1\pi }{50},\frac{0.5\pi }{50}\;,\;\frac{\pi }{50}\;,\frac{2\pi }{50}\;,\frac{3\pi }{50}\;,\frac{5\pi }{50}$, and $\frac{8\pi }{50}$, we evaluate the variations $∆\bar{k}$ by using Equation (9) for each case. The results for $∆\bar{k}$ as a function of σ are given in Figure 10a. From the results, we see that as σ increases, the variation $∆\bar{k}$ initially increases and then reaches the maximum (${\left(∆\bar{k}\right)}_{max}$) at an intermediate value of σ ($\sigma^{*}$). After the maximum, this variation decreases and finally becomes zero at σ = 1. In fact, this behavior of $∆\bar{k}$ with a maximum is similar to the plot by Wu and Lu for the rate constant sensitivity against σ [30].

To better understand the existence of the maximum at an intermediate value of σ, or $\sigma^{*}$, we consider the dependence of the rate constant on the patch arrangement at low and high values of σ. For this analysis, we define the average relative variation per patch, ${∆}_{rel}{\bar{k}}_{1}\left(\sigma \right)$, which is defined as ${\bar{k}}_{max}\left(\sigma \right)/\left({\bar{k}}_{min}\left(\sigma \right)\cdot N\right)$. Here, we note that $∆\bar{k}$ can be expressed in terms of ${∆}_{rel}{\bar{k}}_{1}\left(\sigma \right)$ and ${\bar{k}}_{min}\left(\sigma \right)$, as demonstrated in the following equation:

(10) $$∆\bar{k}\;=\;{\bar{k}}_{max}-\;{\bar{k}}_{min}\;=\;{\bar{k}}_{min}\left(\frac{{\bar{k}}_{max}}{{\bar{k}}_{min}\cdot N}N-1\right)\;=\;{\bar{k}}_{min}\left({∆}_{rel}{\bar{k}}_{1}∆N-1\right)$$

At a low value of σ, the patches can have a large variety of arrangements, since there are many available locations for the patches, and, thus, the patch competitions can be significant (when aggregated) or ignorable (when maximally dispersed). Therefore, ${∆}_{rel}{\bar{k}}_{1}\left(\sigma \right)$ is large. However, $∆\bar{k}$ itself is small, because the magnitude of ${\bar{k}}_{min}$ is small due to a small value of the reactive area fraction σ. In contrast, at a high value of σ, there is not much room for variation in the patch arrangement, because the patches are crowded. Therefore, the patches have a small variety of arrangements, and competition cannot be ignored. Accordingly, ${∆}_{rel}{\bar{k}}_{1}\left(\sigma \right)$ is small, although the magnitude of ${\bar{k}}_{min}$ is large, which is the opposite trend, as in the above case, with a low value of σ. Therefore, as σ increases, ${∆}_{rel}{\bar{k}}_{1}\left(\sigma \right)$ decreases while ${\bar{k}}_{min}\left(\sigma \right)$ increases. From Equation (10), since $∆\bar{k}$ depends on the product of two quantities showing opposite behaviors against σ, or ${\bar{k}}_{min}\cdot {∆}_{rel}{\bar{k}}_{1}$, we understand that the maximum should appear at an intermediate value of σ, as is demonstrated in Figure 10b. In Figure 10b, we display ${\bar{k}}_{min}$, ${∆}_{rel}{\bar{k}}_{1}\left(\sigma \right)$ and $∆\bar{k}$ as functions of σ for the case of $\theta \;=\;\frac{2\pi }{50}$.

Finally, since the value of ${\left(∆\bar{k}\right)}_{max}$ in Figure 10a is dependent on the size of a single patch characterized by θ, we analyze the maximum variation (${\left(∆\bar{k}\right)}_{max}$) and its corresponding σ value ($\sigma^{*}$) as functions of the area fraction of a single patch ($\sigma_{1}$). The results are shown in Figure 10c. As $\sigma_{1}$ (or θ) increases, ${\left(∆\bar{k}\right)}_{max}$ decreases and $\sigma^{*}$ increases, which were also observed in the Wu and Lu study [30]. The trend of ${\left(∆\bar{k}\right)}_{max}$ can be explained by the fact that, as $\sigma_{1}$ decreases, ${\bar{k}}_{max}$ increases and ${\bar{k}}_{min}$ decreases, and $∆\bar{k}$ is thus larger for a fixed value of σ. The trend of $\sigma^{*}$ can be explained by the fact that, as $\sigma_{1}$ increases, the distribution of $∆\bar{k}$ is shifted to the right in the plot of $∆\bar{k}$ versus σ, because the unit patch area $\sigma_{1}$ is larger.

#### 3.4.2. Variation in the Overall Rate Constant in the Lu Reaction Systems

Another interesting case for the variation in the rate constant is a case with a fixed value of σ, where other parameters N and a are free to change under that restriction of σ. However, since we consider identical patches, N and a are directly related, as we discuss in Section 3.3 (Lu reaction systems).

Here, the task of our interest is to obtain a range of the rate constant for a given value of σ using our kinetic theory. For this task, we can use Equation (9). However, it should be noted again that, in Equation (9), N and a are not independent parameters, since they are related by $Na^{2}\;=\;4R^{2}\sigma$. Therefore, using $\frac{Na}{R}\;=\;2\sqrt{N}\sqrt{\sigma }$, we can remove a so that we can obtain the following formulas from Equation (9):

(11) $$∆\bar{k}\;=\;\left(\frac{2\sqrt{N}\sqrt{\sigma }\left(1+\frac{3\left(\pi -2\right)}{2}\sqrt{\sigma }-\left(\pi -2\right)\sigma \right)}{2\sqrt{N}\sqrt{\sigma }\left(1+\frac{3\left(\pi -2\right)}{2}\sqrt{\sigma }-\left(\pi -2\right)\sigma \right)+\pi {\left(1-\sigma \right)}^{2}}\right)\;\;-\left(\frac{2\sqrt{\sigma }\left(1+\frac{3\left(\pi -2\right)}{2}\sqrt{\sigma }-\left(\pi -2\right)\sigma \right)}{2\sqrt{\sigma }\left(1+\frac{3\left(\pi -2\right)}{2}\sqrt{\sigma }-\left(\pi -2\right)\sigma \right)+\pi {\left(1-\sigma \right)}^{2}}\right)$$

In Equation (11), the second term, or the lower bound, is immediately determined once σ is given. However, to determine the first term, or the upper bound, we need to specify the value of N, in addition to σ. That is, in the equation, N is a free parameter without any restrictions. Therefore, N can range from 1 to infinity, or the area of a single patch can range from σ (N = 1) to infinitesimal (N = ∞). If infinitesimal patches are allowed, mathematically, the first term, or the upper bound, in Equation (11) becomes 1. Therefore, for the Lu reaction systems, the variations $∆\bar{k}$ are estimated as follows from Equation (11), respectively:

(12) $$∆\bar{k}\;=\;1-\left(\frac{2\sqrt{\sigma }\left(1+\frac{3\left(\pi -2\right)}{2}\sqrt{\sigma }-\left(\pi -2\right)\sigma \right)}{2\sqrt{\sigma }\left(1+\frac{3\left(\pi -2\right)}{2}\sqrt{\sigma }-\left(\pi -2\right)\sigma \right)+\pi {\left(1-\sigma \right)}^{2}}\right)$$

In fact, for a fixed value of σ, the property that, as N goes to infinity, $\bar{k}$ becomes 1, or $\underset{N\to \infty }{\lim}\bar{k}\;=\;1$, was also previously discussed by Lu [29], using the argument that the quantity Q defined as $\frac{\bar{k}}{1-\bar{k}}$ is proportional to $N^{1/2}$, and, thus, when N goes to infinity, $\bar{k}$ should be 1 for Q to be infinity. However, we can directly show that $\underset{N\to \infty }{\lim}\bar{k}\;=\;1$ from the formulas in Equations (1), (2), (3), (4), and (6) by letting N be infinity.

**Figure 11:**
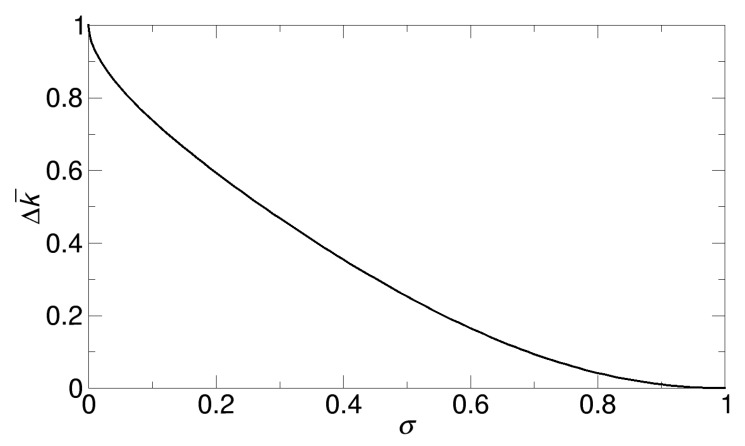
Variations $∆\bar{k}$ calculated from Equation (12) for the Lu reaction systems as a function of the total reactive area fraction σ.

For the Lu reaction systems, we plot the variations $∆\bar{k}$ calculated from Equation (12) as a function of σ shown in Figure 11. In contrast to the cases in Section 3.4.1., where the patch size is fixed, there is no maximum at an intermediate value of σ, and, instead, as σ increases, the variation $∆\bar{k}$ monotonically decreases. This is because the maximum value of $\bar{k}$ is 1 for any value of σ. That is, even in the case of a very small-sized patch, if the single patch is broken into a very large number of smaller patches, $\bar{k}$ becomes 1. However, in reality, a patch would have a physical limit in decreasing its size, and the reactivity of smaller patches could be reduced. 

In this Section 3.4., we show how to calculate the variations $∆\bar{k}$ by estimating the maximum and minimum values of $\bar{k}$ using our kinetic theory. However, since the formulas of our theory provide only a single value of $\bar{k}$ as the maximum value or the ensemble-averaged value of the rate constant, the distribution of the rate constant cannot be obtained, while the distribution can be obtained from BD simulations [26,30]. Therefore, to theoretically obtain the distribution of $\bar{k}$, we may need to develop a new theoretical method to consider an ensemble, as in the BD simulations. This would be a subject of future study.

## 4. Conclusions

In this work, we studied the diffusion-limited (or diffusion-controlled) reaction kinetics of N identical disk-like reactive patches on a sphere that react with partner-reactant molecules around the sphere. Specifically, we comprehensively studied how the overall rate constant is dependent on the patch size, the number of patches, and their spatial arrangement on the sphere. Studies on these characteristic factors of patches have been conducted previously by BD simulations, but the studies were not complete in that they were focused on cases with small-sized patches or with small fractions of the total reactive area. To study complementary cases with large-sized patches or with large fractions, we employed another numerical method called the FEM. From the FEM (large patches) and the previous BD simulation (small patches) results, we evaluated the accuracy of various kinetic theories by comparing the rate constants normalized by the Smoluchowski rate constant, or $\bar{k}$. Here, although $\bar{k}$ in the FEM and kinetic theories is calculated for the most symmetric or uniform patch arrangement and $\bar{k}$ in the BD simulations is the ensemble average for random patch arrangements, a comparison between them was possible, because the value of $\bar{k}$ from a symmetric arrangement is very close to the ensemble-averaged value.

In particular, comparisons were made for two types of model systems; one system (Northrup system) is that the size of patches is fixed, and the other (Lu system) is that the fraction of the total reactive area is fixed. An overall comparison indicates that our kinetic theory based on the curvature-dependent kinetic theory, the LBW theory, and the BH theory gave better agreement with the numerical results than other theories. Given this reliability, we used our kinetic theory to further study the kinetic properties of reactive patches on a sphere. 

Using our formula for multiple patches (N≥1), we investigated the variation of the rate constant under fixed sizes of patches (Northrup system). For a given number N, we estimated the upper and lower bounds of the rate constant. Specifically, since the rate constant obtained from the formula comes from the assumption that patches are evenly distributed (competition is minimized), the rate constant from the formula with N provides the maximum value. On the other hand, when the patches are aggregated as a single larger patch (competition is maximized), the rate constant from the formula with N = 1 provides the minimum value. For the minimum value, we can use the formula for a single patch (N = 1), although the formula for multiple patches with N = 1 also gives an accurate value of rate constant for a single patch.

We also studied the reaction kinetics under fixed values of σ (Lu system) using our formula for multiple patches. As in Lu’s work based on BD simulations [29], we also discussed that, as long as the patch size is infinitely small or the number of patches is infinite, $\bar{k}$ becomes 1, no matter how small σ is. This is physically somewhat surprising, but it is mathematically correct from the formula.

From the above findings, we can conclude that, to increase the rate constant, we divide patches into smaller patches and arrange them in a way that the patches are maximally separated from each other. On the other hand, if we arrange them in a way that they are close to each other or we merge them into larger patches, the rate constant would decrease. Therefore, these findings provide a way to control the reaction kinetics of reactive patches on a sphere and a way to rationally design a reactant with a desired overall reactivity. Physically, these modifications of the arrangement and the size are related to adjusting the strength of competition among the patches.

We also explicitly studied the competition effect under a fixed value of patch size (Northrup-type system). For this study, we considered cases with two, three, and four reactive patches. The rate constants calculated from the FEM for various specific arrangements showed that, as the patches become more separated, the overall rate constant increased. However, our formula has a limitation in that it cannot be applicable to such a calculation of the rate constant for specific patch arrangements, since the formula only provides the maximum or the ensemble-averaged value of the rate constant. Therefore, one future research direction for the improvement of our formula is to take into account the explicit dependence of the rate constant on the patch arrangement, as in the LBW theory [35]. In addition to this, there are still a few theoretical challenges remaining in the development of the kinetic theory: (1) to find the probability distribution function of the rate constant for random patch arrangements and (2) to mathematically rigorously find the patch arrangement that gives the maximum rate constant for an arbitrary number N of patches, although it is expected to be a symmetric arrangement in many cases, which may have a similar level of mathematical difficulty to that in the Tammes problem.

Although the system with identical patches that we considered in this work has been proven as a very useful model to understand the chemical kinetics of patches, there is still room for improvement for more realistic systems. One improvement would be to generalize the model to include patches with different sizes or to consider a heterogeneous mixture of different reactive patches. Another improvement would be to introduce patch-patch interactions in the kinetic model. In reality, this would be an important factor in kinetics, because it would directly affect the patch distribution, resulting in the modification of the overall rate constant. For example, if reactive patches are mobile and may prefer to aggregate, the ensemble-averaged rate constant would be close to the rate constant for a single larger patch, instead of the rate constant for multiple smaller patches with maximal separation from each other. 

## Figures and Tables

**Table 1:** Maximum values of the normalized rate constant $\bar{k}$ from the finite element method (FEM) results (Figure 4), and the calculated values from various theories for N patches on a sphere. To avoid confusion about the maximum values in the FEM results, we round up the values to three decimal points so that the maximum values are the values at ϕ = 180° (N = 2), 120° (N = 3), and 109.5° (N = 4), which correspond to symmetric arrangements of the patches.

Number of Patches N	FEM Result (Maximum Value)	Berg and Purcell (Equation (1))	Zwanzig (Equation (2))	Boundary Homogenization (Equation (3))	LBW (Equation (4))	Ours (Equation (6))
2	0.118	0.100	0.101	0.107	0.123	0.120
3	0.178	0.143	0.146	0.155	0.180	0.177
4	0.232	0.182	0.186	0.200	0.233	0.230

**Figure A1:**
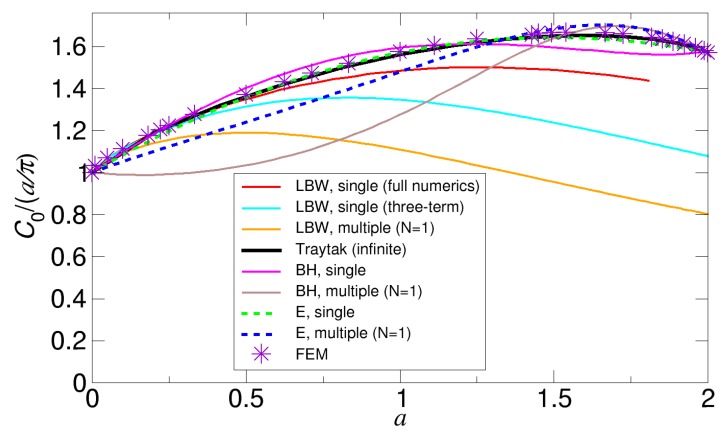
Comparison of the rescaled capacitances $C_{0}/\left(a/\pi \right)$ for a single patch as functions of patch radius a. Figure 5 (a) of Reference [35] provides the results calculated from the LBW formulas for a single patch with an infinite series (LBW, single (full numerics)) and with a finite series up to three terms (LBW, single (three-term)). We also calculate the rescaled capacitance using the formula (Equation (4)) for multiple patches with N = 1 (LBW, multiple (N = 1)). Note that the interaction function ℋ defined in Reference [35], introduced in the derivation of Equation (4), gives 1/8 for N = 1, which is supposed to be zero for N = 1. The Traytak result with infinite-order calculation is from Reference [49]. The results from the BH theory are obtained from the formulas for a single patch and multiple patches given by Equation (A1) (BH, single) and Equation (3) with N = 1 (BH, multiple (N = 1)), respectively. The results from our theory are obtained from Equation (7) (E, single) and Equation (6) with N = 1 (E, multiple (N = 1)), respectively. The FEM results are obtained from Reference [44].

## Appendix A

The Comparison of Rescaled Capacitance from Various Theories for a Single Patch on a Sphere in a Diffusion-Limited Reaction

To evaluate the accuracy of the kinetic theories for a single patch, we perform the comparison of rescaled capacitance $C_{0}/\left(a/\pi \right)$ calculated from the LBW theory for a single patch and multiple patches with N = 1 (Equation (4)) [35], the Traytak theory with an infinite-order calculation [49], the BH theory for a single patch [50] and multiple patches with N = 1 (Equation (3)) [34], our theory for a single patch and multiple patches with N = 1 (Equation (6)) [27], and the FEM result [44]. The BH formula for a single patch [50] is given by:

(A1) $${\bar{k}}_{BH,single}\left(\sigma \right)\;=\;\frac{2\sqrt{\sigma }\left(1+2.6\sqrt{\sigma }-3.2\sigma^{2}\right)}{\pi {\left(1-\sigma \right)}^{3/2}+2\sqrt{\sigma }\left(1+2.6\sqrt{\sigma }-3.2\sigma^{2}\right)}$$

where $\sigma \;=\;\frac{N\pi a^{2}}{4\pi R^{2}}$.

Here, the capacitance $C_{0}$ is defined in Reference [35], and, in this study, the capacitance is numerically equal to the normalized rate constant $\bar{k}$. It should be noted that the Traytak theory with an infinite-order calculation provides the exact value of rate constant and, accordingly, gives the exact value of the rescaled capacitance. The capacitance calculated from various theories are shown in Figure A1.

As one can see in Figure A1, the Traytak result, the results from the BH theory and our theory for a single patch, and the FEM result are very close to each other. However, the LBW results are accurate only when the patch size is small, and they are deviated from the Traytak result as the patch radius a increases. Particularly, the LBW formula for multiple patches, represented by Equation (4), with N = 1 is largely deviated as a increases. This explains why the LBW results give excellent agreement only when a is small (see Figure 9a). In contrast, the BH formula for multiple patches by Equation (3) with N = 1 is more accurate as a increases, and our corresponding formula by Equation (6) with N = 1 generally gives accurate results.
